# Effects of 3d Transition Metal Substitutions on the Phase Stability and Mechanical Properties of Ti–5.5Al–11.8[Mo]_eq_ Alloys

**DOI:** 10.3390/ma16134526

**Published:** 2023-06-22

**Authors:** Naoki Nohira, Keiko Widyanisa, Wan-Ting Chiu, Akira Umise, Masaki Tahara, Hideki Hosoda

**Affiliations:** Institute of Innovative Research (IIR), Tokyo Institute of Technology, 4259 Nagatsuta, Midori-ku, Yokohama 226-8503, Japan

**Keywords:** 3d transition metal, β-stabilizers, Ti alloys, Mo equivalent, phase stability, superelasticity, Ti–Al based alloys

## Abstract

The phase stability, mechanical properties, and functional properties of Ti–5.5Al–11.8[Mo]_eq_ alloys are focused on in this study by substituting 3d transition metal elements (V, Cr, Co, and Ni) for Mo as β-stabilizers to achieve similar β phase stability and room temperature (RT) superelasticity. The ternary alloy systems with the equivalent chemical compositions of Ti–5.5Al–17.7V, Ti–5.5Al–9.5Cr, Ti–5.5Al–7.0Co, and Ti–5.5Al–9.5Ni (mass%) alloys were selected as the target materials based on the Mo equivalent formula, which has been applied for the Ti–5.5Al–11.8Mo alloy in the literature. The fundamental mechanical properties and functionalities of the selected alloys were examined. The β phase was stabilized at RT in all alloys except for the Ti–Al–V alloy. Among all alloys, the Ti–Al–Ni alloy exhibited superelasticity in the cyclic loading–unloading tensile tests at RT. As a result, similar to the Ti–5.5Al–11.8Mo mother alloy, by utilizing the Mo equivalent formula to substitute 3d transition metal elements for Mo, a RT superelasticity was successfully imposed.

## 1. Introduction

The β-titanium (β-Ti) alloys are attractive metallic materials for biomedical applications owing to their excellent biocompatibility [1,2]. In addition, they have also been applied to other applications, such as in aerospace and automobiles, due to their high specific strength, good ductility, and good performance in terms of fatigue [3,4,5]. In other words, the β-Ti alloys not only exhibit high biocompatibility for biomedical applications but also possess sufficient mechanical properties for various applications. In particular, β-Ti-based shape memory alloys (SMAs) have been extensively studied due to their functional properties, such as the shape memory effect (SME) and superelasticity [6,7,8,9,10,11,12,13], which originate from the reversible martensitic transformation (MT) between the parent β phase (bcc) and the α″-martensite phase (orthorhombic) [14,15,16]. This work, hence, focuses on the developments of the β-Ti-based SMAs.

Nevertheless, it is widely known that the practicability of the β-Ti SMAs is constrained by the ω phase, which brings the suppression of MT and brittleness to the β-Ti alloys [17,18,19]. An efficient and facile way to alleviate the formation of the ω phase is thus highly demanded. It has been reported that the addition of a relatively high concentration of tin (Sn) and aluminum (Al) could be an effective strategy to suppress the generation of the athermal ω (ω_ath_) phase, resulting in a cause of MT-inhibition and the isothermal ω (ω_iso_) phase corresponding to the ω embrittlement [20,21,22,23,24]. In addition, our research group also reported that the Ti–Al–Mo ternary system with the nominal chemical composition of Ti–5.5Al–11.8Mo (mass%) achieved a superelastic behavior at room temperature (RT; i.e., 297 K) by introducing a relatively high concentration of Al to suppress the formation of the ω_ath_ phase [25]. This enhancement, namely, achieving superelasticity at RT, was attributed to the suppression of the generation of the undesired ω_ath_ phase via an elevated Al addition concentration. Based on the literature above, in this study, the Al addition amount for suppressing the ω_ath_ phase was determined to be at 5.5 mass%, which is relatively high compared to those in the literature.

Concerning the second additional elements for the manipulation of the alloy functionality by tuning the β phase stability, the 3d transition metals are considered to be promising additive elements due to their light weight, low price, and proper properties based on the screening results [13]. In addition, Duwez et al. also reported the influence of various element additions, including the 3d transition metals, on the alteration of the martensitic transformation start temperature (*M*_s_) of the Ti alloys [26]. For revealing the RT superelasticity, the aforementioned manipulations of the phase stability and phase transformation temperature are critical prerequisites. Those elements, which reduce the *M*_s_ by a small additive amount (i.e., strong β-stabilizer), are favored due to their small influence on the lattice deformation strains. Based on these considerations, vanadium (V), chromium (Cr), cobalt (Co), and nickel (Ni) were chosen in the 3d transition metal group as the second additional elements, making the alloys a ternary system. These elements are typical β-stabilizing elements listed in the often-seen Mo equivalent equations [27] discussed later.

The additive elements were thus determined to be Al and some of the 3d transition metals according to their merits, mentioned above. Furthermore, it is necessary to determine the concentration of the element additions to manipulate the phase stability and tune the phase transformation temperature to impose functionalities, such as RT superelasticity, on the alloys. It has been reported that at RT, the Ti–5.5Al–11.8Mo alloy possesses the metastable β phase, which allows the reversible stress-induced martensitic transformation (SIMT) to be practicable; hence, the exhibition of RT superelasticity was realized. Moreover, alloys that possess the same Mo equivalent ([Mo]_eq_) are expected to exhibit similar phase stabilities (i.e., in this study, the phase stability of the parent β phase in different alloys) [27]. The phase stabilities of β-Ti alloys are often evaluated by using empirical [Mo]_eq_ formulas, quantifying the contribution of each alloying element to the phase stability based on that of the Mo element, which is served as a standard β-stabilizer element. In this work, in view of the phase stability issue, the ternary alloy systems with nominal compositions equal to the Ti–5.5Al–11.8[Mo]_eq_ (mass%) alloys are also expected to exhibit RT superelasticity. The amount of the V, Cr, Co, and Ni elements were calculated to be 17.7, 9.5, 7.0, and 9.5 (mass%), respectively, based on the following [Mo]_eq_ formula [27].

[Mo]_eq_ = [Mo] + 0.67 [V] + 1.25 [Cr] + 1.25 [Ni] + 1.7 [Co] (mass%) (1)

In brief, first, the Ti–Al–Mo alloys were designed for the suppression of the ω_ath_ phase by a relatively high additional amount of Al concentration. Second, some of the appropriate 3d transition metals were chosen for the functionalization (i.e., RT superelasticity) of the alloys. Third, to manipulate the proper phase stability of the parent β phase by tuning the 3d transition metals, the additional amounts of the V, Cr, Co, and Ni elements were determined based on the [Mo]_eq_ formula. Lastly, fundamental investigations, such as the microstructure observations and mechanical property examinations, as well as the evaluations of the functional properties of the Ti–5.5Al–11.8[Mo]_eq_ (mass%) alloys with the additions of the V, Cr, Co, and Ni elements were carried out. It was found that the Ti–5.5Al–9.5Ni alloy, which exhibits a single parent β phase at RT, performed a superelastic behavior at RT.

## 2. Experimental Section

### 2.1. Specimen Fabrications

As mentioned in the introduction section, based on the [Mo]_eq_ formula of (1) [27], the nominal compositions of the alloys in this study were determined to be Ti–5.5Al–17.7V, Ti–5.5Al–9.5Cr, Ti–5.5Al–7.0Co, and Ti–5.5Al–9.5Ni (mass%), respectively, for the purpose of the identical [Mo]_eq_, as shown in Table 1. High-purity Ti (99.99%), Al (99.99%), V (99.9%), Cr (99.99%), Co (99.9%), and Ni (99.99%) metals were used for the preparations of each ternary alloy. The four different ternary alloys were prepared by arc-melting under an Ar–1% H_2_ atmosphere. The small amount of H_2_ in Ar can reduce the oxygen content in Ti-alloy ingots (200–700 ppm), but the residual hydrogen content in the ingots is not increased (~10 ppm). Thus, the effects of O and H on the phase stability and mechanical properties are negligible.

**Figure 1:**
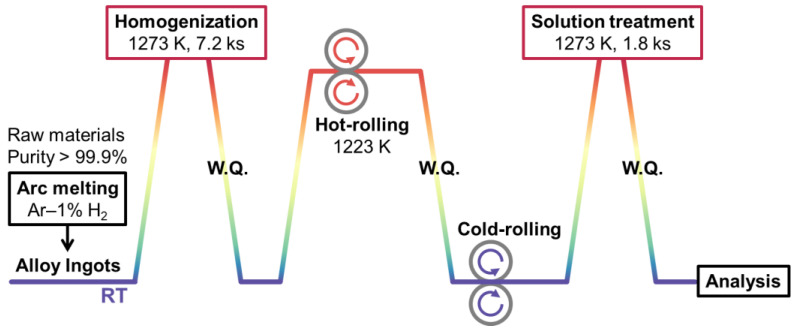
Illustration of overall thermomechanical treatment.

After the arc-melting, the alloy ingots were sealed in an evacuated quartz tube with a vacuum of 4.0 × 10^−4^ Pa, followed by a purge process by inserting Ar gas (purity of 99.9999%). The encapsulated ingots under Ar atmosphere were homogenized at 1273 K for 7.2 ks, followed by an iced-water quenching (W.Q.). The ingots were cleaned and mechanically polished to remove surface contaminations and oxidation layers. The homogenized ingots were then hot-rolled at around 1223 K, followed by a cold-rolling at RT (~297 K). The testing specimens were sliced down from the as-rolled sheets with a certain shape (i.e., dog-bone shape) by using electrical discharge machining (EDM). The shaped specimens were then subjected to a solution treatment at 1273 K for 1.8 ks by using the same techniques as the homogenization process, followed by an iced-water quenching process. The solution-treated (ST) specimens served as the testing samples for the following analysis. The illustration of the above-mentioned thermomechanical treatment is shown in Figure 1.

### 2.2. Analysis

Phase constituents of the alloys were identified by using an X-ray diffractometer (XRD; X’Pert-PRO-MPD, Malvern PANalytical, Malvern, UK) at RT. The scanning range was from 20° to 120° with a scan rate of 0.042° s^−1^ by using a CuKα radiation, where V = 45 kV, I = 40 mA, and λ = 0.15405 nm. A standard silicon plate was used as a reference for correcting external errors. The *CaRIne Crystallography* software created by Cyrille Boudias and Daniel Monceau and the *CellCalc* software created by Hiroyuki Miura were used to determine the lattice parameters of the alloys. A scanning electron microscope (SEM; SU5000, Hitachi, Tokyo, Japan) and optical microscopy (OM; VHX-7000, KEYENCE, Osaka, Japan) were utilized for the observations of microstructures. Prior to the SEM observations, specimens were polished by conducting mechanical and electropolishing until there was a fine surface finish. An electrolyte, composed of 176 mL methanol, 106 mL butanol, and 17.6 mL perchloric acid, was used for this electropolishing. The electropolishing temperature was 233 K, and the voltage and current were at 16.4 V and 46 mA, respectively.

Vickers hardness of the alloys was evaluated by using a micro-Vickers hardness tester (HM-102, Mitutoyo, Kawasaki, Japan) at RT. In addition, the mechanical properties were measured by two different tensile tests. The first one was in the manner of continuous tensile mode, and the second one was in the manner of cyclic loading–unloading tensile tests. The continuous tensile test was conducted until the fracture of the specimens. On the other hand, in the cyclic loading–unloading tensile tests, 1% strain was applied per cycle, and the cycles were repeated until the fracture of the specimens or 10% of the overall strain. Both of the tensile tests were conducted by using a universal testing machine (Autograph AG-X plus 5kN, Shimadzu, Kyoto, Japan) at RT with a strain rate of ~8.3 × 10^−4^ s^−1^. The specimen used for the tensile test was in a dog-bone shape with a gauge dimension of approximately 10 mm (length) × 2.28 mm (width) × 0.3 mm (thickness).

## 3. Results and Discussion

### 3.1. Workability

The alloy workability via both hot- and cold-rolling processes is summarized in Table 2. *R*_H_ indicates the percentage of thickness reduction after the specimens were subjected to the hot-rolling process, while, *R*_C_ indicates that after the cold-rolling process. All alloys showed an obvious thickness reduction after hot rolling. The (a) Ti–Al–V alloy showed an *R*_H_ of 76%, while the (b) Ti–Al–Cr, (c) Ti–Al–Co, and (d) Ti–Al–Ni alloys were sufficiently hot-rolled up to an *R*_H_ of greater than 90%. In addition to the evaluations of *R*_H_, the alloys were classified into three groups based on the evaluation of their workability. First, the (b) Ti–Al–Cr and (c) Ti–Al–Co alloys performed good workability. Second, the (d) Ti–Al–Ni alloy was partially cracked at the edges, showing moderate workability. Third, the (a) Ti–Al–V alloy fractured during hot-rolling, indicating the poorest workability among all the alloys. Since the Ti–Al–V alloy could not be processed by both hot- and cold-rolling, some of the evaluations of the Ti–Al–V specimen, such as the mechanical properties, were not conducted in the following sections.

### 3.2. Phase Constitutions

**Figure 2:**
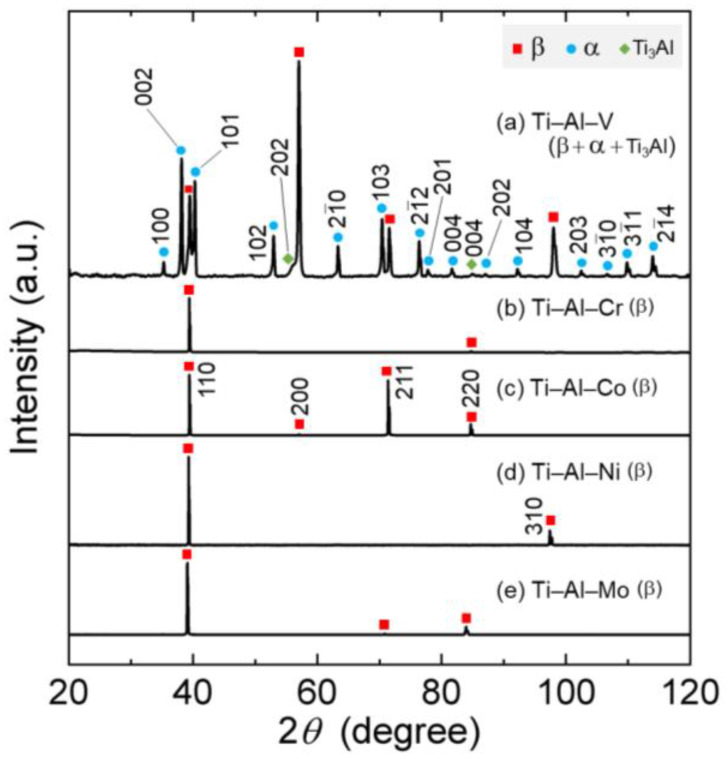
XRD profiles of (a) Ti–Al–V, (b) Ti–Al–Cr, (c) Ti–Al–Co, (d) Ti–Al–Ni, and (e) Ti–Al–Mo [25].

The phase constituents of the alloys were identified by using X-ray diffraction measurements at RT. The XRD profiles of all the alloys are shown in Figure 2. The results indicate that the Ti–Al–X (X = (b) Cr, (c) Co, and (d) Ni) alloys were composed of the single parent β phase. On the other hand, various characteristic peaks besides the parent β phase were observed in the (a) Ti–Al–V alloy. The phase constituents of the (a) Ti–Al–V alloy were verified to be a mixture of β+α+Ti_3_Al phases. Additionally, it is found that the phase constituent of the Ti–Al–V alloy in this work is in accordance with that in the literature [28]. In particular, at around 1223 K, where the alloy was hot-rolled, is the boundary of the region of β/α+β/α_2_ (Ti_3_Al)+β/α+α_2_+β when the V amount is around 13 mass%. At higher V contents (i.e., V > 13 mass%), the phase region of α_2_+β extends, indicating that Ti_3_Al is more likely to be formed. The lattice parameters of all alloys were calculated and are shown in Table 3. Judging from the calculation results, the lattice parameters of the β phase were almost the same among all alloys.

### 3.3. Microstructures

**Figure 3:**
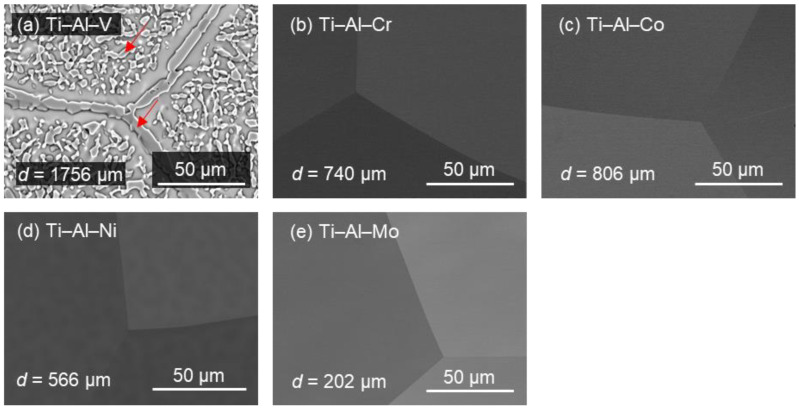
SEM images of (**a**) Ti–Al–V, (**b**) Ti–Al–Cr, (**c**) Ti–Al–Co, (**d**) Ti–Al–Ni, and (**e**) Ti–Al–Mo [25]. The grain size *d* of each alloy is also shown (left-bottom corner).

SEM images of the specimens are shown in Figure 3. All alloys except for the (a) Ti–Al–V alloy show equiaxed grains. Along with the phase identification results from Figure 2, it could thus be determined that the alloys (b) Ti–Al–Cr, (c) Ti–Al–Co, and (d) Ti–Al–Ni were composed of the single parent β phase. Additionally, these microstructures are similar to that of the Ti–Al–Mo alloy in our previous result [25], which is cited in Figure 3e for comparison. On the other hand, in Figure 3a, obvious precipitates, which are pointed out by arrows, can be observed. Those precipitates are formed along the grain boundaries of equiaxed grains or within the grains. The apparent secondary or ternary phase observed in the SEM image is in good agreement with that in the XRD pattern of Figure 2a. According to the XRD pattern, the great amount of precipitates corresponds to the Ti_3_Al compound, which is recognized as a brittle phase [29]. It was thus rational to deduce that the great amount of precipitates could be the reason for the low workability of the (a) Ti–Al–V alloy, as shown in Table 2. The average grain sizes of the equiaxed β grains, which were calculated based on the OM images (not shown), are also indicated as *d* at the left-bottom corners in Figure 3. It is found that the grain sizes differ from each other, indicating that the recrystallization behavior during thermal treatment after cold rolling could be attributed to the different elements introduced, such as Cr, Co, Ni, and Mo.

### 3.4. Vickers Hardness

**Figure 4:**
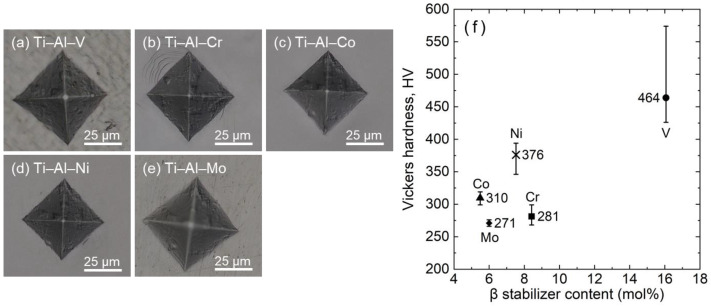
Optical micrographs of Vickers indentation in (**a**) Ti–Al–V, (**b**) Ti–Al–Cr, (**c**) Ti–Al–Co, (**d**) Ti–Al–Ni, and (**e**) Ti–Al–Mo alloys, as well as (**f**) the Vickers hardness of each alloy.

The OM images (Figure 4a–e) show a typical diamond shape after micro-indentation tests using a Vickers hardness tester at RT. It was found that the deviations of the Vickers hardness of the (b) Ti–Al–Cr, (c) Ti–Al–Co, (d) Ti–Al–Ni, and (e) Ti–Al–Mo alloys are relatively small, while the deviation of the (a) Ti–Al–V alloy is comparatively large (Figure 4f). This is due to the triple-phase-composed alloy being relatively not evenly distributed compared to the single-β-composed phase alloys (please see Figure 2 for the phase constituents and Figure 3 for the microstructures). Moreover, it is clear that the hardness of the (a) Ti–Al–V alloy is higher than that of the other alloys. This could be attributed to the precipitation hardening of the triple-phase (i.e., the β + α + Ti_3_Al phases)-composed Ti–Al–V alloy. Meanwhile, the Vickers hardness for the other alloys is close to each other (Figure 4f). Apart from the effect of the precipitates, the hardness of metastable β-Ti alloys is discussed by the solid-solution strengthening effect and also by the stability of the β phase [30,31], respectively, as described below. First, the alloys exhibiting a single β phase, namely, the (b) Ti–Al–Cr, (c) Ti–Al–Co, (d) Ti–Al–Ni, and (e) Ti–Al–Mo alloys, have almost identical phase stabilities because of equal Mo equivalents. Second, however, these alloys possess different molar concentrations of each additional element. Therefore, the amount of solid-solution strengthening differs for each alloy. The Vickers hardness of these alloys is close (see the left-bottom corner of Figure 4f), but the trend is difficult to be determined. The β-Ti–4 mol% Au-based SMAs alloyed with 5 mol% 3d transition elements of Cr, Co, Ni, and Mo specimens were examined in the previous study [13]. First, the Ni-introduced alloy was classified as the highest hardness group, possessing a hardness of about 400 HV. Second, the Co-introduced alloy was classified as the second highest group with a hardness of about 350 HV. Lastly, the Cr or Mo-introduced alloys were in the third hardest group, ranging from 270 to 300 HV. This is similar to the hardness trend in this study. In addition, the range of hardness in this study is also similar to those in the literature.

### 3.5. Tensile Tests

**Figure 5:**
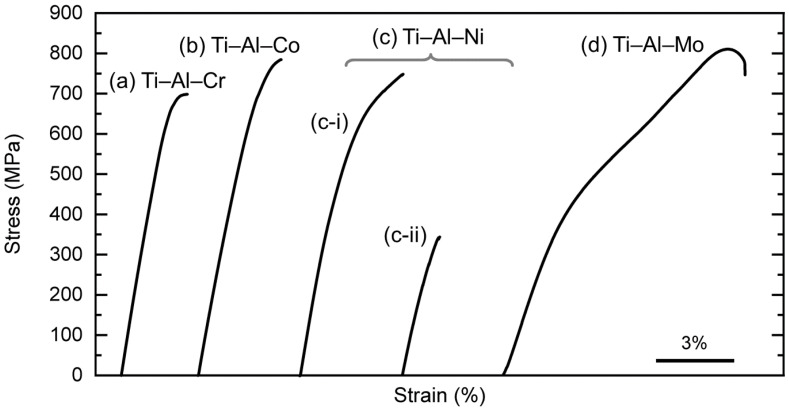
Stress–strain curves obtained from continuous tensile tests of (a) Ti–Al–Cr, (b) Ti–Al–Co, (c) Ti–Al–Ni, and (d) Ti–Al–Mo [25] alloys at RT. (c) Ti–Al–Ni alloy had deviations in deformation behavior, so the representative ones are shown as (c-i,c-ii).

**Figure 6:**
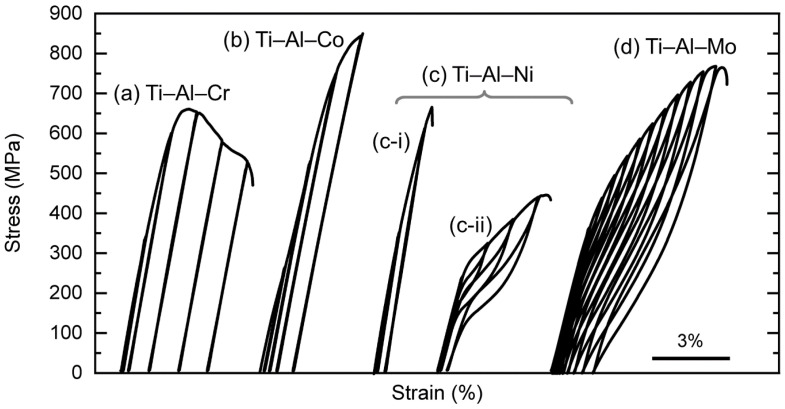
Stress–strain curves obtained from cyclic loading–unloading tensile tests of (a) Ti–Al–Cr, (b) Ti–Al–Co, (c) Ti–Al–Ni, and (d) Ti–Al–Mo [25] alloys at RT. (c) Ti–Al–Ni alloy had deviations in deformation behavior, so the representative ones are shown as (c-i,c-ii).

As mentioned in the Experimental Section, tensile tests were conducted in two different manners at RT in this study. The continuous tensile tests and the cyclic loading–unloading tensile tests are shown in Figure 5 and Figure 6, respectively. It is necessary to mention that since the Ti–Al–V alloy was not able to be rolled, both the tensile tests were performed without the Ti–Al–V specimen.

In the continuous tensile tests, it was found that no obvious two-stage yielding behavior could be found in the Ti–Al–X (X = (a) Cr, (b) Co, and (c) Ni) alloys. On the other hand, it was found that the Ti–Al–Mo [25] alloy shows slight two-stage yielding in the literature. The first-stage yielding indicates the stress-induced martensite transformation (SIMT), while the second yielding suggests the plastic deformation (i.e., slip of dislocations). As a result, the elongation of the (d) Ti–Al–Mo alloy is superior to others. The (c) Ti–Al–Ni alloy showed deviations in its deformation behavior. The representative results are shown in Figure 5c-i,c-ii. As mentioned in Section 3.1, the Ti–Al–Ni alloy was partially cracked after the rolling process, which may have led to the formation of microcracks inside the material, resulting in fractures during elastic deformation such as in Figure 5c-ii. Hereafter, (c-i) is treated as the appropriate deformation behavior of the Ti–Al–Ni alloy. Most of the alloys in this study fractured at around 2–4% of the overall applied strain, while only the (c) Ti–Al–Ni alloy yielded slightly. This indicates that the alloys in this study did not undergo the SIMT during the continuous tensile tests or only underwent a certain limited SIMT. The mechanical properties, such as strengths (i.e., 0.2% stress and ultimate tensile strength (UTS)) and elongation, which were read from the continuous tensile tests in Figure 5, are summarized in Table 4.

In the cyclic loading–unloading tensile tests (Figure 6), the (c-ii) Ti–Al–Ni alloy showed a superelasticity similar to that of the (d) Ti–Al–Mo alloy [25]. The (d) Ti–Al–Mo alloy performed the best elongation with a total strain of about 7%, while the elongation of the (c-ii) Ti–Al–Ni alloy was approximately 4%. On the other hand, no obvious superelasticity could be found in the (a) Ti–Al–Cr and (b) Ti–Al–Co alloys. This could be attributed to whether the SIMT did not occur during the loading process or the high reverse martensitic transformation start temperature (*A*_s_).

In the (c) Ti–Al–Ni alloy, the results of the cyclic tensile tests also showed deviations in the deformation behavior. Some specimens, such as (c-i), had high yield stress and did not show superelasticity. Two possibilities could be responsible for these differences in deformation behavior: one is crystallographic orientation dependence, and the other is compositional variation. However, compositional variation was not a likely cause in this study. The results of the XRD measurements (Figure 2) and microstructure observations (Figure 3) indicate no precipitates nor a second phase in the Ti–Al–Ni alloy that would alter the composition of the β phase. Moreover, the XRD peaks of the β phase are broad when compositional variations occur, which was also not observed in this study. On the other hand, it is known that the SIMT depends on crystal orientation; the grain size of Ti–Al–Ni is more than 500 μm (Figure 3d), and only a few grains exist in the gauge of the specimen at most. Therefore, the deformation behavior may change significantly when the texture is different. The following discusses the superelastic behavior of the Ti–Al–Ni alloy shown in Figure 6c-ii.

**Figure 7:**
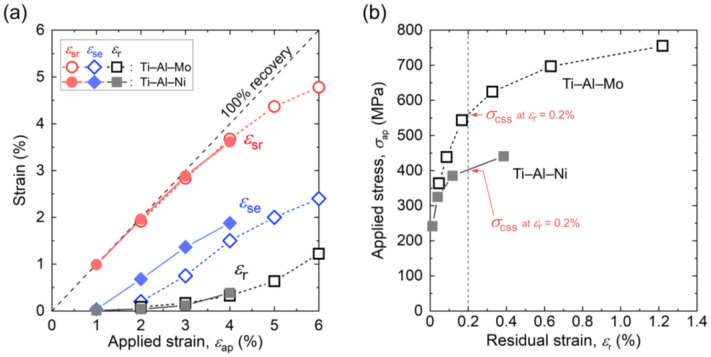
(**a**) Total shape recovery strain (*ε*_sr_), superelastic recovery strain (*ε*_se_), and residual strain (*ε*_r_) read from cyclic loading–unloading tensile tests of the Ti–Al–Ni alloy (solid symbols) and the Ti–Al–Mo alloy (open symbols). The dotted diagonal line represents 100% shape recovery during unloading. (**b**) Relationship between applied stress (*σ*_ap_) and residual strain (*ε*_r_) per cycle.

To evaluate the superelastic properties of the (c) Ti–Al–Ni and (d) Ti–Al–Mo [25] alloys that exhibited RT superelasticity, the overall shape recovery strain *ε*_sr_ (including elastic recovery strain), superelastic recovery strain *ε*_se_ (excluding elastic recovery strain), and residual plastic strain *ε*_r_ of each cycle read from the cyclic stress–strain curves (Figure 6) are plotted as a functions of applied strain *ε*_ap_ in Figure 7a. Because of the poor ductility of the Ti–Al–Ni alloy, plots present up to 4% of the applied strain.

In both alloys, with increasing *ε*_ap_, the *ε*_sr_ and *ε*_se_ increased monotonically. The *ε*_sr_ indicates that the Ti–Al–Mo alloy and Ti–Al–Ni alloy perform similar overall shape recovery behavior at 4% of the applied strain, showing a maximum *ε*_sr_ of about 3.6% for the Ti–Al–Ni alloy and an *ε*_sr_ of a little bit higher than 3.6% for the Ti–Al–Mo alloy. Meanwhile, the maximum *ε*_se_ for the Ti–Al–Ni alloy was 1.9%. This is considered to be relatively small, showing about half of the recently developed alloys with a maximum *ε*_se_ of 4% [24] due to the relatively poor elongation of this specimen. The *ε*_r_ results of the Ti–Al–Mo and Ti–Al–Ni alloys are also similar.

To explain the deformation behaviors of these two alloys, the maximum applied stress (*σ*_ap_) and the *ε*_r_ were read from the stress–strain curves (Figure 6), and their correlation is shown in Figure 7b. Intersections of the dashed lines and curves in Figure 7b indicate each critical stress for the slip (*σ*_CSS_), which refers to the stress when *ε*_r_ is at 0.2%. According to Figure 7b, the *σ*_CSS_ is 402 MPa for the Ti–Al–Ni alloy, while it is 565 MPa for the Ti–Al–Mo alloy. Given that the *σ*_CSS_ of the Ti–Al–Mo alloy is higher than that of the Ti–Al–Ni alloy, the residual strain, which is a consequence of the introduced dislocations, is more easily introduced into the Ti–Al–Ni alloy than the Ti–Al–Mo alloy.

In Ti–Al–Ni alloys, further strengthening and improvement of the ductility of the alloy are required. However, it is worth mentioning that based on the superelastic Ti–Al–Mo alloy, this study successfully imposed RT superelasticity on the Ti–Al–Ni alloy by using the Mo equivalent formula. A further improvement of the Ti–Al–Ni alloy can be achieved by certain thermomechanical treatments and the fine-tuning of the alloy composition.

In brief, in this study, RT superelasticity was achieved in a newly developed Ti–Al–Ni alloy because of two successful strategies. The first strategy was to design a relatively high Al concentration to suppress the ω_ath_ phase, and the second one was to use the [Mo]_eq_ equation to manipulate the appropriate phase stability of the parent β phase by tuning the 3d transition metal as a third element. As a result, in the Ti–Al–Ni alloy, the SIMT between the β phase and the αʺ-martensite phase and its reverse transformation took place at RT, resulting in the achievement of RT superelasticity.

## 4. Conclusions

Ti–5.5Al–X (X = V, Cr, Co, and Ni) (mass%) alloys were prepared in this study, and additive amounts of the 3d transition metals were determined according to the Mo equivalent ([Mo]_eq_) formula. Some findings, which could be a guideline for the development of the Ti–5.5Al-based alloys, are listed as follows:

1. Good workability, except for the Ti–Al–V alloy, was achieved in the Ti–Al–X (X = Cr, Co, and Ni) alloys.
2. Via the phase identification by using X-ray measurements, the Ti–Al–V alloy showed a triple phase of β+α+Ti_3_Al, while other alloys were composed of the single parent β phase.
3. In agreement with the phase identification, the microstructures also displayed a single β phase in the Ti–Al–X (X = Cr, Co, and Ni) alloys, while a multi-phase was found in the Ti–Al–V alloy.
4. High Vickers hardness with a relatively large deviation was found in the triple-phase-composed Ti–Al–V alloy, while that of other alloys was comparatively low.
5. Superelastic behavior at room temperature was successfully imposed on the Ti–5.5Al–9.5Ni alloy (mass%), which showed a shape recovery of about 3.6% and a superelastic recovery of 1.9%.

## Figures and Tables

**Table 1:** Nominal composition of the alloys based on the [Mo]_eq_ formula of (1) and the Ti–5.5Al–11.8Mo alloy in [25].

Ti–5.5Al–X Alloy	Mo [25]	(a) V	(b) Cr	(c) Co	(d) Ni
mass% X	11.8	17.7	9.5	7.0	9.5
mol% X	6.0	16.1	8.4	5.5	7.5

**Table 2:** Workability of (a) Ti–Al–V, (b) Ti–Al–Cr, (c) Ti–Al–Co, and (d) Ti–Al–Ni alloys after rolling processes.

Alloy	Hot-Rolling (*R*_H_)	Cold-Rolling (*R*_C_)	Condition
(a) Ti–Al–V	76%	×	Fractured
(b) Ti–Al–Cr	94%	29%	Good
(c) Ti–Al–Co	91%	52%	Good
(d) Ti–Al–Ni	91%	34%	Partially cracked

**Table 3:** Lattice parameters of β phase (*a*_β_) in (a) Ti–Al–V, (b) Ti–Al–Cr, (c) Ti–Al–Co, (d) Ti–Al–Ni, and (e) Ti–Al–Mo [25] alloys.

Alloy	Lattice Parameter, *a*_β_ (nm)	Standard Deviation
(a) Ti–Al–V	0.3226	0.00579
(b) Ti–Al–Cr	0.3234	0.00037
(c) Ti–Al–Co	0.3233	0.00157
(d) Ti–Al–Ni	0.3239	0.00087
(e) Ti–Al–Mo [25]	0.3252	0.00087

**Table 4:** Mechanical properties of Ti–Al–X (X = (a) V, (b) Cr, (c) Co, (d) Ni, and (e) Mo [25]) alloys.

	(a) V	(b) Cr	(c) Co	(d) Ni	(e) Mo [25]
**0.2% stress, *σ*_y_** **(MPa)**	-	675	661	420	372
**UTS, *σ*_UTS_** **(MPa)**	-	711	791	748	810
**Fracture strain** **(%)**	-	2.6	3.3	4.1	9.5

## Data Availability

Data is contained within the article.
